# West Nile Virus in Mexico: Evidence of Widespread Circulation since July 2002.

**DOI:** 10.3201/eid0912.030564

**Published:** 2003-12

**Authors:** José G. Estrada-Franco, Roberto Navarro-Lopez, David W.C. Beasley, Lark Coffey, Anne-Sophie Carrara, Amelia Travassos da Rosa, Tamara Clements, Eryu Wang, George V. Ludwig, Arturo Campomanes Cortes, Pedro Paz Ramirez, Robert B. Tesh, Alan D.T. Barrett, Scott C. Weaver

**Affiliations:** * University of Texas Medical Branch, Galveston, Texas, USA; † Comision Mexico-Estados Unidos para la Prevencion de la Fiebre Aftosa y Otras Enfermedades Exoticas de los Animales, Mexico City, Mexico; ‡ U.S. Army Medical Research Institute of Infectious Diseases, Fort Detrick, Maryland, USA

**Keywords:** West Nile virus, arbovirus, flavivirus, equine, phylogeny, serology

## Abstract

West Nile virus (WNV) antibodies were detected in horses from five Mexican states, and WNV was isolated from a Common Raven in the state of Tabasco. Phylogenetic studies indicate that this isolate, the first from Mexico, is related to strains from the central United States but has a relatively high degree of sequence divergence.

During the summer of 2002, the Agricultural Ministry of Mexico (SAGARPA) received reports of encephalitis-like illness in horses from several different areas of Mexico, concurrent with reports of West Nile virus (WNV) encephalitis outbreaks in horses along the Texas border in the states of Coahuila, Tamaulipas, and Chihuahua. Other suspected cases were reported from several southern, tropical states. We report the results of an equine serosurvey conducted from July 2002 to March 2003 by the Office of Exotic Diseases of the Agricultural Ministry (CPA-SAGARPA). We also describe the first isolation of WNV in Mexico, in a Common Raven (*Corvus corax*) from the state of Tabasco.

The Republic of Mexico is divided by the Tropic of Cancer, with temperate, arid climate zones in the north and at higher elevations and humid, subtropical, and tropical climate zones in the south. Our study encompassed most of these climatic zones, as equine serum samples were collected from 3 border states, 1 state on the Tropic of Cancer, and 10 states south of the Tropic (Figure 1). Sampled equine populations were chosen on the basis of a history of clinical encephalitis; medical history was provided by owners and corroborated by CPA-SAGARPA veterinarians. In total, 441 serum samples were analyzed for WNV antibodies.

**Figure 1:**
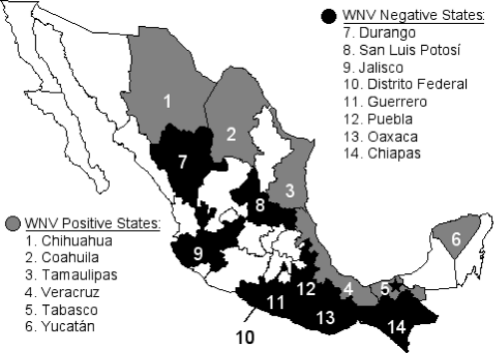
Map showing the Mexican states sampled for antibodies to West Nile virus and Venezuelan equine encephalitis virus in equines. Unshaded states were not sampled. The location of the West Nile virus isolation from a dead Common Raven is shown by a star.

Because most serum samples were collected late in the probable virus transmission season, all were first screened for immunoglobulin (Ig) G antibodies, using IgG enzyme-linked immunosorbent assays (ELISA) with a recombinant, envelope protein domain III antigen expressed and purified from Escherichia coli (D.W.C. Beasley, et al., submitted for pub.). Positive samples were confirmed by hemagglutination inhibition (HI) tests against WNV and St. Louis encephalitis virus (SLEV), by 90% plaque reduction neutralization tests (PRNT) against WNV (*1*), and by ELISA with WNV, SLEV, and Venezuelan equine encephalitis virus (VEEV) antigens and viruses. (The presence of several endemic arboviruses, including SLEV and VEEV, necessitated additional testing.) WNV infection was confirmed if the WNV antibody titer was >fourfold higher than the SLEV titer. To investigate evidence of recent WNV infection, 198 samples were also tested by using both IgG- and IgM-specific ELISA with WNV-infected cell culture antigens (*2*). Selected samples were tested by ELISA and PRNT for VEEV antibodies to determine if this virus was circulating in areas reporting equine encephalitis.

## Results

A total of 441 equine serum samples from 14 states of Mexico were tested (Figure 1). WNV-specific antibodies were detected in 97 (22%) of the samples. These data probably overestimate the true equine seropositivity rate because sampling focused on herds with a history of clinical encephalitis. Representative data from 22 of the WNV-positive samples obtained in five different states are presented in Table 1. No evidence was obtained of SLEV infection in equines, but VEEV-specific antibodies were detected in serum samples from the states of Veracruz and Yucatán. (Horses are vaccinated against VEEV in the states of Chiapas and Oaxaca, so samples from these locations were not tested for VEEV antibodies.) The positive samples, several of which also contained WNV IgG, represent natural VEEV circulation and infection of horses, presumably with enzootic subtype IE strains (*3*,*4*).

**Table 1:** Serologic analysis^a^ of 22 horse serum samples from five Mexican states, positive for West Nile virus antibodies, July 2002–March 2003^b^

					WNV serologic findings							HI SLEV	
State, locality	Sample no.	Date	Age	Sex	IgG-E	IgM	IgG		PRNT	HI		
Veracruz, Minatitlan	VER-015	Oct 28, 2002	7 y	M	pos	1,600	400		>320	20	20	
Veracruz, Minatitlan	VER-011	Oct 28, 2002	18 m	M	pos	neg	400		>320	160	neg	
Veracruz, Hidalgotitlan	VER-017	Oct 29, 2002	8 m	M	pos	12,800	neg		>320	>640	160	
Veracruz, Texistepec	VER-26	Oct 29, 2002	2 y	M	pos	neg	100		40	160	neg	
Veracruz, Texistepec	VER-025	Nov 7, 2002	18 m	M	pos	6,400	neg		>320	>640	160	
Veracruz, J. Carranza	VER-036	Nov 11, 2002	7 m	M	pos	neg	6,400		>320	>640	40	
Veracruz, Angel R Cabada	406	Mar 20, 2003	13 m	F	pos	neg		400	>320	80	neg			
Veracruz, Cosamaloapan	187	Aug 28, 2002	5 y	F	pos	neg		1,600	>320	160	20			
Yucatan, Merida	389	Mar 7, 2003	4 y	F	pos	neg		1,600	nt	80	20			
Yucatan, Tizimin	391	Mar 1, 2003	5 y	M	pos	neg		1,600	160	40	20			
Chihuahua, Ojinaga	210	Oct 20, 2002	2 y	M	neg	1,600		neg	40	320	40			
Coahuila, Cd Acuna	112	Oct 14, 2002	3 y	M	pos	1,600		neg	80	20	20			
Coahuila, Hidalgo	4	Sep 1, 2002	5	F	pos	400		100	>320	>640	20			
Coahuila, Villa Union	26	Nov 2, 2002	4 y	M	pos	400		100	>320	320	80			
Coahuila, Nava	67	Jul 11, 2002	7 y	M	pos	400		100	>320	320	20			
Coahuila, P. Negras	56	Nov 5, 2002	2 y	M	pos	6,400		100	>320	>640	40			
Coahuila, Zaragoza	39	Oct 10, 2002	5 y	F	pos	neg		400	>160	320	20			
Coahuila, Morelos	51	Oct 5, 2002	7 y	M	pos	neg		100	>160	160	40			
Tamaulipas, Diaz Ordaz	268	Nov 7, 2002	8 y	F	pos	12,800		1,600	40	320	40			
Tamaulipas, Camargo	279	Nov 11, 2002	4 y	F	pos	12,800		3,200	>320	320	80			
Tamaulipas, Rio Bravo	349	Nov 5, 2002	8 y	M	pos	neg		400	80	80	neg			
Tamaulipas, Victoria	344	Nov 7, 2002	2 y	M	pos	neg		6,400	>320	160	20			

^a^Titers expressed as reciprocal of dilution; all tests were enzyme-linked immunosorbent assays unless otherwise noted; all tests were against WNV (West Nile virus) unless otherwise noted. ^b^SLEV, St. Louis encephalitis virus; Ig, immunoglobin; G-E, recombinant domain III of E protein; PRNT, plaque reduction neutralization test; HI, hemagglutination inhibition test.

**Figure 2:**
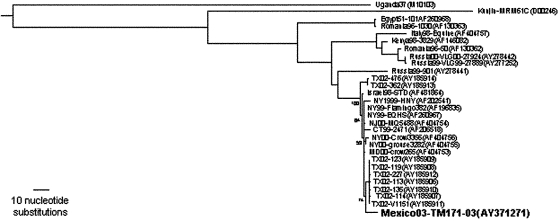
Phylogenetic tree, derived by maximum likelihood (ML) by using prM-E sequences for West Nile virus (WNV) isolates including the 2003 Mexican Raven isolate. Strains are indicated by country or abbreviated state (U.S.) followed by year and strain designation. GenBank accession nos. are in parentheses. A lineage 2 WNV strain (AY277251) was used to root the tree. Numbers indicate bootstrap values from 1,000 replicates.

On May 5, 2003, the CPA-SAGARPA received a report of a dead Common Raven from the El Yumka wildlife preserve in the city of Villahermosa, state of Tabasco. Although this species is native to Tabasco and other regions of Mexico, this bird was one of two ravens imported from the United States in 1999. A necropsy was performed, and virus isolation was attempted on tissue samples at the CPA-SAGARPA biosafety level 3 facility in Palo Alto, Mexico City. On May 16, 2003, cytopathic effects were detected in Vero cells injected with brain suspension. Viral RNA from the isolate was genetically characterized at the National Institute for Epidemiology and Diagnostics (InDre) in Mexico City and at the University of Texas Medical Branch in Galveston. A 2,004-nt genome portion, including the prM-E protein region, was amplified by using a reverse transcription–polymerase chain reaction as described previously (*5*); the resulting amplicons were sequenced directly with the Big Dye sequencing kit and model 3100 sequencer (Applied Biosystems, Foster City, CA). The sequence of this WNV isolate (TM171-03, submitted to GenBank under accession no. AY371271) was aligned with all homologous WNV sequences of the same length available from the GenBank library (homologous to nt 466–2,469 in the Flamingo382 strain, GenBank accession no. AF196835), and phylogenetic trees were constructed by using maximum parsimony, maximum likelihood (incorporating empirical base frequencies, a general time-reversible substitution model with the following frequencies: A→C 1.34263; A→G 4.18575; A→U 1.55497; C→G 0.044980; C→T 15.08737; G→U 1.00000, a γ shape parameter of 0.228), and neighbor joining programs implemented in the PAUP 4.0 software package (*6*). All trees had nearly identical branching orders; the maximum parsimony tree is presented in Figure 2. All trees placed the Mexican raven isolate as a sister to a clade that comprises most WNV strains isolated in Texas during 2002. Two other WNV strains from the Bolivar Peninsula, Texas (362, 476), were positioned basally to a 1998 Israeli stork isolate that grouped with all other North American isolates, suggesting that the North American strains may not all have originated from a point source introduction into the New York area in 1999. This topologic finding was the result of a single synonymous, third codon position (genomic position 969 by using the numbering of the Flamingo382 isolate, GenBank accession no. AF196835) U nucleotide (synapomorphy) shared by the 1998 Israeli and all North American strains except the Bolivar Peninsula isolates. However, the relatively poor support, represented by bootstrap values (1,000 replicates) of only 56–59 by using the three different phylogenetic methods, suggests that this topologic finding is not necessarily correct. More robust phylogenetic analyses using complete genomic sequences are needed to clarify these relationships.

Comparison of nucleotide sequences indicated mutations at 9 nt (0.45%) of the Mexican TM171-03 sequence compared to the prototype NY99 strain (Flamingo382, GenBank accession no. AF196835). Some of these nucleotide positions vary among other strains sampled worldwide, suggesting that they are not under strong purifying selection. Comparison with sequences of Year 2002 Texas strains showed only one shared mutation (genomic position 2466 C→U). WNV has remained genetically conserved in the New World; however, based on our limited sequencing, the Mexican strain appears to be the most divergent WNV isolate identified to date in the Americas (Table 2). Two of the mutations resulted in amino acid changes at prM141 (Ile→Thr) and E156 (Ser→Pro), which have not been reported to date in North American isolates. The Ser→Pro amino acid substitution at residue E156 is of interest, as it abolishes a potential glycosylation site that is a putative WNV virulence determinant (*7*–*9*). This report is the first of a New World WNV isolate with probable altered E protein glycosylation. Further studies are in progress to assess the possible phenotypic effects of this mutation.

**Table 2:** Nucleotide and deduced amino acid differences in the prM-E gene region between the prototype New York (382-99) and Mexican (TM171-03) West Nile virus strains

Nucleotide (amino acid)^a^	Strain 382-99	Strain TM171-03	
483	C	U	
858	C	U	
887 (prM141)	U (Ile)	C (Thr)	
1137	C	U	
1432 (E156)	U (Ser)	C (Pro)	
1626	C	U	
2328	C	U	
2388	C	U	
2466	C	U	

^a^Nucleotide numbering used for the New York flamingo 382-99 sequence (Genbank accession no. AF196835); locations of encoded amino acid differences are shown in parentheses.

## Conclusions

Two recent publications reported serologic evidence of WNV infection among equines in the states of Yucatán and Coahuila from serum samples collected in October and December 2002, respectively (*10*,*11*). We obtained serologic evidence of earlier and more widespread circulation of WNV in five other Mexican states, dating back to July 2002. We also report the first isolation of WNV from Mexico from a dead Common Raven that resided in a wildlife preserve in Tabasco.

Genetic studies indicated that the Mexican WNV strain was likely introduced from the central United States. The level of genetic divergence (9 nt) of the Mexican isolate and the unique amino acid substitutions in the prM and E proteins when compared to all other North American WNV isolates suggest that the Mexican strain has been evolving independently for some time and did not simply enter Mexico recently from Texas. We speculate that this strain descended from a WNV strain introduced into the Yucatán peninsula by migrating birds. Nucleotide sequences of viruses isolated from Mexican states close to the U.S. border, once obtained, may more closely resemble strains isolated in Texas during 2002.

Of particular interest is the overlap in distribution of WNV and VEEV (serologic data for VEEV not shown) in the southern Mexican states of Veracruz and Yucatán; the presence of other flaviviruses like SLEV in these states is also likely. Both WVN and VEEV produce clinically similar neurologic disease in horses, and past, presumptive diagnoses of VEEV may have been inaccurate. Steps are now in place at the Mexico City headquarters of the Animal Health Division of SAGARPA to implement appropriate laboratory diagnosis for flaviviruses and alphaviruses. Additionally, field personnel are instructed to investigate epidemiologic signs of possible WNV infection including avian death and unusual human neurologic syndromes.

The biologic and epidemiologic consequences of mosquito-borne encephalitis viruses (*12*) cocirculating in the same ecosystem should be examined. The impact of WNV on human health in regions (such as Mexico) where inhabitants may have extensive prior exposure to other flaviviruses such as dengue, SLEV, Ilheus, Bussuquara, Jutiapa, and Yellow fever viruses may differ from that in regions (e.g., the United States and Canada) where human exposure to flaviviruses is very limited. WNV infection in persons with previous flavivirus immunity, which could either attenuate disease because of cross-protective antibodies (*13*) or potentially worsen disease because of immune enhancement (*14*), should be studied. Our ongoing VEEV surveillance in southern Mexico may identify differences in transmission habitats for VEEV and WNV and assist with optimizing virus containment efforts.
